# Perioperative Predictors of Early Recurrence for Resectable and Borderline-Resectable Pancreatic Cancer

**DOI:** 10.3390/cancers13102285

**Published:** 2021-05-11

**Authors:** Masafumi Imamura, Minoru Nagayama, Daisuke Kyuno, Shigenori Ota, Takeshi Murakami, Akina Kimura, Hiroshi Yamaguchi, Toru Kato, Yasutoshi Kimura, Ichiro Takemasa

**Affiliations:** Department of Surgery, Surgical Oncology and Science, Sapporo Medical University, Sapporo 060-8543, Japan; mnagayam@sapmed.ac.jp (M.N.); kyuno@sapmed.ac.jp (D.K.); oshige@sapmed.ac.jp (S.O.); murakami.takeshi@sapmed.ac.jp (T.M.); akina030@sapmed.ac.jp (A.K.); hyama@sapmed.ac.jp (H.Y.); toru.kato1969@gmail.com (T.K.); kimuray@sapmed.ac.jp (Y.K.); itakemasa@sapmed.ac.jp (I.T.)

**Keywords:** carbohydrate antigen 19-9, early recurrence, pancreatic ductal adenocarcinoma, postrecurrence survival

## Abstract

**Simple Summary:**

Most patients with a pancreatic ductal adenocarcinoma develop a recurrence after surgery. Predictive factors may therefore guide therapeutic decision-making. We aimed to identify perioperative predictors of the early recurrence of pancreatic ductal adenocarcinomas. We found that preoperative (>52 U/mL) and postoperative (>37 U/mL) elevated carbohydrate antigen 19-9 levels as well as a tumor size >3.0 cm were independently associated with an early recurrence after a pancreatectomy. Furthermore, an early recurrence resulted in a more frequent liver metastasis than a late recurrence, suggesting that patients experiencing a recurrence within 12 months had undetectable micrometastases. Further studies are needed to identify new biomarkers for the detection of clinically occult micrometastases during surgery as current preoperative risk factors are inadequate to accurately identify patients susceptible to an early recurrence of pancreatic ductal adenocarcinomas.

**Abstract:**

We aimed to identify the perioperative predictors of the early recurrence (ER) of resectable and borderline-resectable pancreatic ductal adenocarcinomas (PDACs). After surgery for a PDAC, most patients develop a recurrence. Predictive factors may therefore guide therapeutic decision-making. Patients (*n* = 234) who underwent a pancreatectomy for a PDAC between 2006 and 2019 were included. The postrecurrence survival (PRS) was estimated using Kaplan–Meier curves. Predictive factors for an ER were assessed using logistic regression analyses; 93 patients (39.7%) were recurrence-free at the last follow-up. Patients with an ER (*n* = 85, 36.3%), defined as a recurrence within the first 12 months after surgery, had 1- and 2-year PRS rates of 38.7% and 9.5%, respectively, compared with 66.9% and 37.2% for those with a late recurrence (*n* = 56, 23.9%; both *p* < 0.001). The most common site of an ER was the liver (55.3%) with a significantly shorter median overall survival time than that with either a local or a lung recurrence (14.5 months; *p* < 0.001). Preoperative and postoperative risk factors for an ER included a tumor size >3.0 cm (odds ratio (OR): 3.11, 95% confidence interval (CI): 1.35–7.14) and preoperative carbohydrate antigen 19-9 (CA19-9) levels >52 U/mL (OR: 3.25, 95% CI: 1.67–6.30) and a pathological tumor size >3.0 cm (OR: 2.00, 95% CI: 1.03–3.90) and postoperative carbohydrate antigen 19-9 levels >37 U/mL (OR: 2.11, 95% CI: 1.02–4.36), respectively. Preoperatively (>52 U/mL) and postoperatively (>37 U/mL) elevated CA19-9 and a tumor size >3.0 cm were independent predictors for an ER after a pancreatectomy for a PDAC.

## 1. Introduction

A pancreatic ductal adenocarcinoma (PDAC), one of the most aggressive cancers worldwide, is predicted to become the second leading cause of cancer-related deaths in Western countries within the next 10 years [1]. In Japan, a PDAC is the fourth leading cause of cancer-related deaths; the number of patients is predicted to increase in the future [2]. A complete tumor resection remains the only potentially curative option for a PDAC. The criteria for resectability have been proposed by the National Comprehensive Cancer Network (NCCN) [3]. Recommended therapeutic strategies are applied according to this classification to improve prognosis; however, even in cases classified as resectable according to the NCCN guidelines, a low postoperative survival rate has been reported [4,5]. Moreover, approximately 80% of patients experience local and metastatic recurrence, with >50% occurring within the first 12 months after curative surgery [6].

Recurrence within the first 12 months after surgery is considered to be an “early recurrence” (ER) and is a characteristic of a PDAC. The perioperative predictors of an ER such as the tumor size [6,7,8], metastases in the harvested lymph nodes [6,7,8], the serum carbohydrate antigen 19-9 (CA19-9) value [6,9,10,11,12], the duration of symptoms [13], a modified Glasgow Prognostic Score [14], a Charlson age-comorbidity index [6], tumor differentiation [6,12,13] and p53 expression in the primary tumor [15] have been reported to identify high-risk patients. Recently, neoadjuvant therapy has been shown to provide oncological benefits more than upfront surgery in patients with a borderline-resectable PDAC (BR-PDAC) [16,17]; significant survival benefits of neoadjuvant chemotherapy have also been demonstrated in phase III of the study on patients with a resectable PDAC (R-PDAC) [18].

Currently, more PDAC patients are being treated with neoadjuvant therapy prior to curative surgery; however, most of the above reports on perioperative predictors were studies involving an R-PDAC. These studies do not mention the association between resectability or neoadjuvant therapy and an ER; therefore, it is necessary to identify risk factors for an ER while taking these into consideration. Potential predictive factors for a postoperative ER may guide decision-making to extend the neoadjuvant therapy duration as well as the choice of adjuvant therapy.

The purpose of this study was to identify perioperative predictors of an ER of an R- and BR-PDAC. We focused on the time of recurrence, patterns of recurrence, postrecurrence survival (PRS) and perioperative clinicopathological factors for an ER after a curative resection.

## 2. Materials and Methods

### 2.1. Study Population and Inclusion and Exclusion Criteria 

Between January 2006 and December 2019, 265 consecutive patients underwent a pancreatic resection for a pancreatic adenocarcinoma at Sapporo Medical University Hospital (Sapporo, Japan). All patients were histologically diagnosed with a ductal adenocarcinoma of the pancreas confirmed by two pathologists. The exclusion criteria were as follows: an intraductal papillary mucinous carcinoma or a pancreatic intraepithelial neoplasia, incomplete records owing to follow-up at other institutions or <12 months of follow-up. Of the 265 patients, 31 were excluded owing to conversion surgery for an unresectable cancer (*n* = 23), a re-pancreatectomy for recurrence in the remnant pancreas after an initial pancreatectomy (*n* = 6) and 30-day postoperative mortality (*n* = 2). Data of the remaining 234 patients were retrospectively analyzed. All 234 patients included in this study were preoperatively diagnosed with an R-or BR-PDAC according to the NCCN guidelines (version 1, 2019) [3] during a multidisciplinary team meeting.

### 2.2. Outcome Measures

Data on preoperative and postoperative demographics as well as clinicopathological and treatment characteristics were extracted from a prospectively maintained institutional database. Preoperative and postoperative CA19-9 levels were obtained, when available. CA19-9 measurements were performed within one month before surgery; CA19-9 levels acquired at a time of jaundice (total bilirubin > 5 mg/dL) or later than two months postoperatively were excluded from the analysis. Furthermore, CA19-9 levels < 3 U/mL were related to Lewis-negative patients [19] and were also excluded from the analysis.

The preoperative tumor diameter was measured using endoscopic ultrasonography both at the first examination and prior to neoadjuvant therapy [20]. The surgical procedure involved a standard or subtotal stomach- or pylorus-preserving pancreatoduodenectomy in 155 (66.3%), a distal pancreatectomy in 74 (31.6%) and a total pancreatectomy in 5 (2.1%) patients. A regional lymph node dissection was performed on all patients; resected specimens were fixed in 10% formalin at room temperature. The size and gross appearance of the tumor were recorded. The pathological stage of all tumor specimens was determined according to the Union for International Cancer Control Tumor–Node–Metastasis (TNM) staging system (eighth edition) [21]. The tumor status following surgery was defined using the residual tumor (R) classification: R0, no residual tumor; R1, a microscopic residual tumor; R2, a macroscopic residual tumor.

### 2.3. Neoadjuvant/Adjuvant Therapy and Follow-Up

Neoadjuvant therapy has been administered to R- and BR-PDAC patients since 2008 exclusively when the patients voluntarily registered for the clinical trial at the time and their respective attending physicians obtained informed consent from them. Neoadjuvant therapy involved chemoradiotherapy in 25 (10.7%), nab-paclitaxel plus gemcitabine in 20 (8.5%) and S-1 in 9 (3.8%) patients. Adjuvant therapy has been administered postoperatively to most patients since 2006. Adjuvant therapy involved gemcitabine according to the results of the CONKO-001 trial [22]. S-1 has been administered since 2013 according to the results of the JASPAC01 trial [23].

The patient follow-up was performed by either a surgical, medical or radiation oncologist at the outpatient clinic of our hospital or affiliated hospitals. In general, an enhanced computed tomography (CT) of the chest, abdomen and pelvis was performed every three months within the first year postoperatively; after one year, an enhanced CT was performed every six months for another five years. If elevated CA19-9 levels were observed preoperatively, then this was re-evaluated every three months. Magnetic resonance imaging and/or ^18^F-fluorodeoxyglucose positron emission tomography/CT were performed, if necessary, to clarify ambiguous CT findings. 

When imaging findings were consistent with a recurrence, only the first site was documented. The recurrence sites were stratified into six mutually exclusive categories: liver, lung, peritoneum, remnant pancreas, local and other. Local recurrence was defined as recurrence within the surgical area such as the soft tissue along the aorta, superior mesenteric or celiac artery or around the hepaticojejunostomy or pancreatojejunostomy site. Other was defined as recurrence at other, less common sites. An ER was defined as a recurrence within the first 12 months after surgery in this study, as a previous study concluded that a recurrence-free interval of 12 months was the optimal threshold for differentiating between an ER and a late recurrence (LR) [6].

### 2.4. Statistical Analyses

The clinicopathological features were compared between patients who experienced a recurrence within the first year and those who did not. The categorical variables were compared using the χ^2^ test while the continuous variables were compared using a Student’s *t*-test or the Mann–Whitney *U* test. A receiver operating characteristic (ROC) curve was constructed to estimate the optimal cut-off value for the preoperative serum CA19-9 level as a risk factor for an ER; this was determined to be the point on the ROC curve closest to the upper-left corner of the graph. Associations between potential risk factors and an ER were assessed using a univariate logistic regression analysis. Variables with a *p*-value < 0.05 were included as covariates in two separate multivariate logistic regression analyses: one for preoperative and one for postoperative risk factors associated with an ER.

The results are presented as odds ratios (ORs) with corresponding 95% confidence intervals (CIs). Overall survival (OS) was defined as the time from surgery to either death or the last follow-up. PRS was defined as the time from the first recurrence to either death or the last follow-up. Survival was calculated using the Kaplan–Meier method and compared between groups using the log-rank test; a two-sided *p*-value < 0.05 was considered statistically significant. Statistical analyses were performed using BellCurve for Excel (version 3.21; Social Survey Research Information Co., Ltd., Tokyo, Japan).

## 3. Results

### 3.1. Cohort Characteristics

During the study period, a total of 234 patients comprising 121 men (51.7%) and 113 women (48.3%) underwent curative intent surgery for a newly diagnosed R- or BR-PDAC. At the diagnosis, the cancer was R- in 171 patients (73.1%), BR-portal vein (PV) in 43 patients (18.4%) and BR-artery (A) in 20 patients (8.5%). The demographic, clinicopathological and treatment characteristics of the entire study population dichotomized by the presence or absence of recurrence are summarized in Table 1. The average age ± standard deviation (SD) was 69.3 ± 9.0 years. All patients underwent an oncological pancreatic resection. Depending on the location and extent of the tumor, a pancreatoduodenectomy (*n* = 155), distal pancreatectomy (*n* = 74) or total pancreatectomy (*n* = 5) was performed. Vascular resections were performed in 82 patients (35.0%). Of the 82 patients, 70 underwent only vein resection, 10 underwent only artery resection and 2 underwent artery and vein resection combined. The tumor status following surgery was as follows: R0 in 220 patients (94.0%), R1 in 13 patients (5.6%) and R2 in 1 patient (0.6%). No significant differences were observed among the R status groups with respect to sex, the American Society of Anesthesiologist physical status (ASA PS) classification system, the body mass index (BMI), the surgical procedure, vascular resection, the histological type or the T-stage. The median preoperative serum CA19-9 level of 203 patients was 59.9 U/mL. A ROC curve demonstrated that a preoperative serum CA19-9 value of 52 U/mL was the optimal cut-off point for an ER after surgery with a sensitivity and specificity of 72.5% and 55.5%, respectively; the area under the ROC curve (AUC) was 0.6630.

Among all patients, 55 and 204 received neoadjuvant and adjuvant therapy, respectively. The median follow-up period was 25.2 (interquartile range, 13.8–43.4) months. At the last follow-up, a recurrence was documented for 141 patients (60.3%); 93 patients (39.7%) exhibited no signs of a recurrence after surgery. Significant differences between the recurrence and non-recurrence groups were observed in the age (*p* = 0.0165), resectability (*p* = 0.0116), preoperative CA19-9 level (*p* < 0.001), preoperative and pathological tumor size (*p* = 0.0018), positive lymph nodes (*p* < 0.001), microscopic perineural and lymphovascular invasion (*p* < 0.001), TNM stage (*p* < 0.001) and adjuvant therapy (*p* = 0.0012).

**Figure 1:**
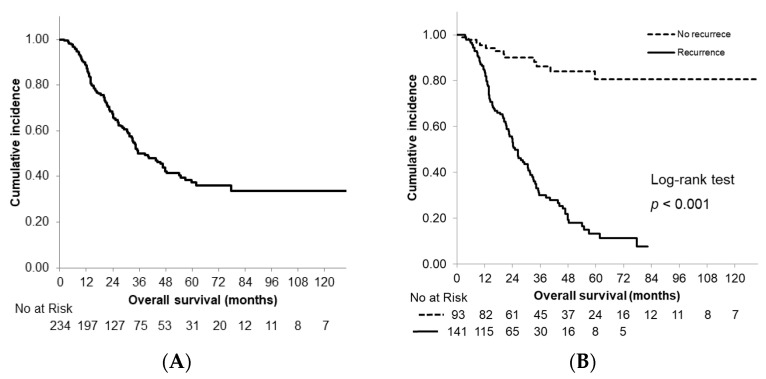
Kaplan–Meier curves of overall survival. (**A**) All patients; (**B**) patients stratified according to recurrence status.

The median OS time for all patients was 38.5 months. The estimated 3- and 5-year survival rates were 50.2% and 37.3%, respectively (Figure 1A). The median OS time in the recurrence group was 25.2 months; by comparison, survival did not reach the median time in the non-recurrence group with a significant difference observed between the groups (*p* < 0.001). The actual 3- and 5-year survival rates in the recurrence group were 30.0% and 13.3%, respectively (Figure 1B).

### 3.2. Postrecurrence Survival between Early and Late Recurrence

Among the 141 patients with a recurrence after surgery, an ER was documented in 85 patients (60.3%) and an LR in 56 patients (39.7%). Patients with an ER tended to exhibit more poorly differentiated tumors as well as a microscopic lymphovascular invasion; additionally, postoperative CA19-9 levels were significantly higher (Table 2). Conversely, there were no significant differences in resectability, tumor size, TNM stage and neoadjuvant/adjuvant therapy between the ER and LR groups.

**Figure 2:**
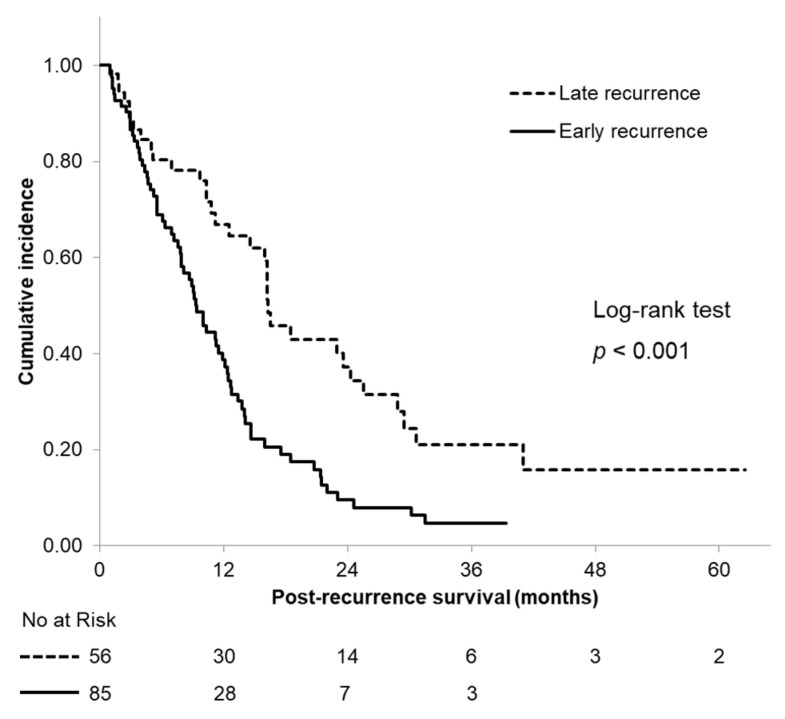
Kaplan–Meier curves of postrecurrence survival according to the time of recurrence.

The median PRS time was significantly longer in patients with an LR (16.3 months, 95% CI: 14.0–18.6) than in those with an ER (9.3 months, 95% CI: 7.7–10.9) (*p* < 0.001; Figure 2). The ER group had 1- and 2-year PRS rates of 38.7% and 9.5%, compared with 66.9% and 37.2% in the LR group, respectively (both *p* < 0.001).

### 3.3. Patterns of Early and Late Recurrence after Surgery

**Figure 3:**
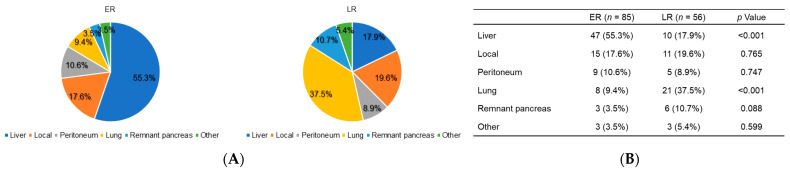
(**A**) Proportion of first recurrence sites and (**B**) comparison of the recurrence patterns at different time points.

The proportion and comparison of recurrence sites at different time points are shown in Figure 3A,B. The recurrence patterns were defined at the first recurrence location. These showed that in the ER group, 47 patients (55.3%) initially exhibited a liver metastasis while 15 patients (17.6%) initially exhibited a local recurrence. Conversely, in the LR group, 21 patients (37.5%) had lung metastases while 11 patients (19.6%) had a local recurrence. The ER group experienced significantly more frequent liver metastases than the LR group whereas the LR group experienced significantly more frequent lung metastases.

### 3.4. Survival Analysis According to the Site of First Recurrence

**Figure 4:**
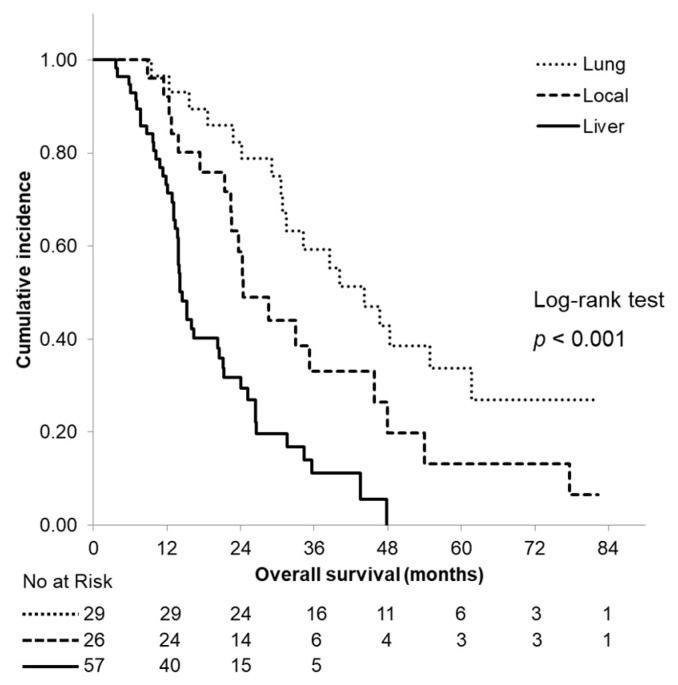
Kaplan–Meier curves of overall survival according to the site of the first recurrence. The median overall survival time was 14.5 months, 24.4 months and 44.2 months in patients who initially exhibited a liver metastasis, a local recurrence and a lung metastasis, respectively.

OS curves according to the site of the first recurrence are shown in Figure 4. Among all of the patients with a recurrence, 57 patients initially exhibited a liver metastasis, 29 patients initially exhibited a lung recurrence and 26 patients initially exhibited a local recurrence. The median OS time was 14.5 months in patients who initially exhibited a liver metastasis and 24.4 and 44.2 months in patients who initially exhibited a local recurrence and a lung metastasis, respectively; a significant difference was observed between the groups (*p* < 0.001). 

### 3.5. Risk Factors Associated with Early Recurrence

The results of two separate univariate and multivariate logistic regression analyses of preoperative and postoperative risk factors are presented in Table 3. Two preoperative variables proved to be independently associated with a recurrence within 12 months after surgery: a preoperative tumor size > 3.0 cm on endoscopic ultrasonography at the first examination (OR: 3.11, 95% CI: 1.35–7.14; *p* = 0.0076) and a preoperative CA19-9 level > 52 U/mL (OR: 3.25, 95% CI: 1.67–6.30; *p* < 0.001). Two postoperative risk factors were independently correlated with an ER including a pathological tumor size > 3.0 cm (OR: 2.00, 95% CI: 1.03–3.90; *p* = 0.0420) and a postoperative CA19-9 level > 37 U/mL (OR: 2.11, 95% CI: 1.02–4.36; *p* = 0.0444). Conversely, both neoadjuvant and adjuvant therapy were not found to be independently associated with a reduction in an ER.

## 4. Discussion

The present study indicated that the median OS of patients with an R- and BR-PDAC was 38.5 months; 60.3% of these patients experienced a recurrence (both late and early) after curative surgery. The median OS of the recurrent cases was 25.2 months; the 5-year survival rate was 13.3%. The proportion of patients with an ER after a curative resection for an R- and BR-PDAC was 36.3%; the median PRS time was 9.3 months, which was significantly shorter than that of patients with an LR (16.3 months; *p* < 0.001). The most common site of an ER was the liver (55.3%), which was observed more frequently than for an LR (17.9%; *p* < 0.001). Moreover, there were significant differences in OS between a liver, local and lung recurrence; patients with a liver recurrence had a significantly shorter median OS time (14.5 months; *p* < 0.001). These results suggested that even if patients with an R- and BR-PDAC could be treated with curative surgery after neoadjuvant therapy, >50% of them would experience a recurrence; additionally, their prognosis would be poor, especially in cases of an ER in the liver.

Recent studies [4,5] reported that even in the most favorable cohort (patients with an R-PDAC), up to 80% of patients experienced a recurrence after a short recurrence-free interval. Another study [5] reported the liver to be the most common site of the first recurrence with a particularly low recurrence-free survival time with a median of 6.9 months. Nevertheless, there is some evidence to suggest that different sites of a recurrence have different survival rates [24,25,26]. Groot et al. [6] reported that patients with a liver recurrence had a worse survival than patients with a local or pulmonary recurrence. Interestingly, metachronous lung metastases as the first and only type of recurrence were found to develop later and had a better OS than presentations of metastatic disease [27,28]. These findings may suggest that patients with an ER in the liver had occult micrometastases that were undetectable using existing imaging modalities, suggesting the insufficiency of effective neoadjuvant therapies at the time of the resection.

In this study, several independent preoperative and postoperative factors associated with an increased likelihood of an ER after surgery for a PDAC were identified including preoperative and postoperative CA19-9 levels and tumor size. CA19-9 was first discovered in 1979 [29] and has become the most clinically useful and well-known biomarker for a PDAC. The CA19-9 level may be a reliable prognostic marker for survival, recurrence and tumor resectability [30,31]. Several reports have established an association between elevated preoperative and postoperative CA19-9 levels and a poor survival after a pancreatectomy for an R-PDAC, suggesting thresholds from 37 to 400 U/mL [32,33,34,35]. However, there are far fewer studies focusing on the correlation between the CA19-9 level and the ER of a PDAC (R- and BR-PDAC) and there is currently no consensus regarding the CA19-9 threshold for predicting an ER.

Groot et al. [6] reported that the optimal preoperative and postoperative CA19-9 cut-off values for predicting a recurrence within 12 months in patients with an R-PDAC (*n* = 957) were > 210 and > 37 U/mL, respectively. Nishio et al. [12] found that for an R-PDAC (*n* = 90), a preoperative CA19-9 level > 529 U/mL was an independent predictive factor for a recurrence within 12 months. Conversely, Tsai et al. [36] reported that following neoadjuvant therapy for an R- and BR-PDAC (*n* = 131), the normalization of CA19-9 levels was the strongest prognostic marker for a long-term survival. 

In this study, the analysis of the ROC curve and the associated AUC values revealed that the optimal preoperative CA19-9 threshold for the prediction of an ER was >52 U/mL; however, with an AUC of 0.663, sensitivity of 72% and specificity of 55%, the predictive strength of an elevated preoperative CA19-9 level was fairly limited, highlighting the necessity of identifying more accurate biomarkers in patients with a PDAC. 

Several methods targeting tumor associated molecules and genes have been investigated to detect an early postoperative recurrence of pancreatic cancer. The target specimens are divided into two main categories: preoperative and postoperative blood samples and resected tissues. Many research findings have been published regarding the association between tumor markers, blood counts and biochemical substances from peripheral blood and the early detection of a metastasis. Circulating nucleic acids and tumor cells are now considered to be additional targets for measurement. Circulating tumor cells (CTCs) are known to be important mediators for the development of metastases; their presence has been demonstrated in several malignancies including pancreatic, colorectal, gastric, ovarian, breast, prostate, bladder, renal and lung cancers [37]. The results of two large meta-analyses [38,39] demonstrated a correlation between CTC positivity and poor outcomes in patients with pancreatic cancer; these analyses concluded that CTCs strongly predicted the disease course in patients with pancreatic cancer, indicating a poor OS and recurrence-free survival. 

In a prospective longitudinal study (CLUSTER trial) including patients with pancreatic cancer [40], the number of CTCs decreased after neoadjuvant chemotherapy and surgery in patients with pancreatic cancer; patients who developed an ER within 12 months postoperatively had a significantly higher preoperative and postoperative CTC [41].

Studies using tumor tissues have reported an association between the ER of pancreatic cancer and the presence of cancer stem cells and novel substances found in omics studies. CD44 is one such marker of cancer stem cells and CD44-positive tumor cells were shown to be associated with tumor initiation, metastasis and prognosis [42]. In pancreatic cancer tissue, CD44 expression could therefore predict an ER [43].

Rho guanine nucleotide factor 2 (*ARHGEF2*, also known as *GEF-H1*) was extracted from a public database as the gene associated with an ER of pancreatic cancer [44]. In a mass spectrometry-based study [45], galectin 4, a group of carbohydrate-binding proteins involved in neoplastic development and progression, was identified as a down-regulated protein in short-term survivors of pancreatic cancer and correlated with an ER [46]. Genome-wide DNA methylation screening revealed that three CpG marker sites including a CpG site in CDK14 could predict an ER in formalin-fixed, paraffin-embedded surgically resected tissue [47].

The results of this study suggested that large tumor diameters or high CA19-9 levels, even after neoadjuvant therapy, may lead to an ER after a pancreatectomy. However, existing clinical parameters alone are insufficient to predict an ER after a pancreatectomy and it is necessary to apply the so-called “liquid biopsy” such as an analysis of CTCs to clinical trials to predict occult micrometastases. The accurate preoperative identification of patients with a high likelihood of an ER will be beneficial for the patients and help the clinicians in decision-making regarding the extension of the neoadjuvant therapy duration as well as the choice of adjuvant therapy for a PDAC.

This study has several limitations worthy of consideration. First, this was a retrospective study conducted at a single institution with a relatively small sample size. As is the case with retrospective studies, the present study consisted of accurate and sufficient data to perform the analysis with adequate precision; however, there were a few missing elements that could not be retrospectively analyzed. Second, approximately 5–10% of the general population were Lewis antigen A- and B-negative, meaning they were unable to synthesize the CA19-9 antigen and could therefore not exhibit elevated CA19-9 levels even in cases of pancreatic cancer. Finally, neoadjuvant therapy includes a variety of regimens reflecting the adaptation of the patients to clinical trials at the time and this variation may have resulted in a degree of error due to a variance in the analysis.

## 5. Conclusions

This study found that preoperatively (>52 U/mL) and postoperatively (>37 U/mL) elevated CA19-9 levels as well as a tumor size > 3.0 cm were independently associated with an ER after a pancreatectomy for both an R- and BR-PDAC. Furthermore, an ER resulted in a more frequent liver metastasis than an LR, suggesting that patients experiencing a recurrence within 12 months had undetected micrometastases. Further studies are needed to identify new biomarkers for the detection of a clinically occult micrometastatic disease at the time of operation as the currently acknowledged preoperative risk factors are inadequate to accurately identify patients susceptible to an ER of a PDAC.

## Figures and Tables

**Table 1:** Demographic, clinicopathological and treatment characteristics of included patients.

Variable	All Patients (*n* = 234)	Non-Recurrence Group (*n* = 93)	Recurrence Group (*n* = 141)	*p*-Value
Male, *n* (%)	121 (51.7)	48 (51.6)	73 (51.8)	0.981
Age, years, mean (SD)	69.3 (9.0)	71.0 (8.5)	68.2 (9.1)	0.0165
ASA PS, *n* (%)				0.9066
1–2	212 (90.6)	84 (90.3)	128 (90.8)	
3	22 (9.4)	9 (9.7)	13 (9.2)	
BMI, kg/m^2^, mean (SD)	22.3 (3.4)	22.1 (3.1)	22.5 (3.6)	0.4416
Resectability, *n* (%)				0.0116
R	171 (73.1)	73 (78.5)	98 (69.5)	
BR-PV	43 (18.4)	9 (9.7)	34 (24.1)	
BR-A	20 (8.5)	11 (11.8)	9 (6.4)	
Preoperative CA19-9 level (U/mL) *				
Median (IQR)	59.9 (21.7–212.1)	31 (12.2–88.8)	106 (31.8–278.8)	<0.001
Postoperative CA19-9 level (U/mL) *				
Median (IQR)	16.6 (8.5–38.9)	13.9 (7.4–30.1)	17.4 (9.1–52.7)	0.0504
Surgical procedure, *n* (%)				0.3059
PPPD	41 (17.5)	18 (19.4)	23 (16.3)	
SSPPD/Std. PD	114 (48.8)	45 (48.4)	69 (48.9)	
DP	74 (31.6)	30 (32.3)	44 (31.2)	
TP	5 (2.1)	0 (0.0)	5 (3.5)	
Vascular resection, *n* (%)	82 (35.0)	28 (30.1)	54 (38.3)	0.1988
Residual tumor, *n* (%)				
R0	220 (94.0)	91 (97.8)	129 (91.5)	0.0844
R1/2	14 (6.0)	2 (2.2)	12 (8.5)	
Histological type, *n* (%)				0.1272
Well-mod. adenocarcinoma	198 (84.6)	84 (90.3)	114 (80.9)	
Poor adenocarcinoma	19 (8.1)	4 (4.3)	15 (10.6)	
Other ^†^	17 (7.3)	5 (5.4)	12 (8.5)	
Tumor size, cm, mean (SD) ^‡^	2.3 (0.9)	2.1 (0.8)	2.5 (0.9)	0.0018
Pathological tumor size, cm, mean (SD)	3.0 (1.4)	2.6 (1.1)	3.3 (1.6)	<0.001
T-stage, *n* (%)				0.2096
0–2	195 (83.3)	81 (87.1)	114 (80.9)	
3	39 (16.7)	12 (12.9)	27 (19.1)	
Positive lymph nodes, *n* (%)	149 (63.7)	43 (46.2)	106 (75.2)	<0.001
Perineural invasion, *n* (%)	200 (85.5)	69 (74.2)	131 (92.9)	<0.001
Lymphovascular invasion, *n* (%)	131 (56.0)	38 (40.9)	93 (66.0)	<0.001
Venous invasion, *n* (%)	153 (65.4)	54 (58.1)	99 (70.2)	0.0559
TNM stage, *n* (%)				<0.001
0–1	77 (32.9)	45 (48.4)	32 (22.7)	
2	89 (38.0)	38 (40.9)	51 (36.2)	
3	47 (20.1)	7 (7.5)	40 (28.3)	
4	21 (9.0)	3 (3.2)	18 (12.8)	
Neoadjuvant therapy, *n* (%)				0.7019
None	179 (76.5)	69 (74.2)	110 (78.0)	
Chemotherapy	30 (12.8)	14 (15.1)	16 (11.3)	
Chemoradiotherapy	25 (10.7)	10 (10.7)	15 (10.6)	
Adjuvant therapy, *n* (%)				0.0012
None	30 (12.8)	21 (22.6)	9 (6.4)	
Chemotherapy	200 (85.5)	70 (75.3)	130 (92.2)	
Chemoradiotherapy	4 (1.7)	2 (2.2)	2 (1.4)	

* Patients (*n* = 203) had perioperative CA19-9 measurements available. Patients (*n* = 31) with CA19-9 levels < 3 U/mL (related to Lewis-negative patients) were excluded. ^†^ Other consisted of histologic types such as adenosquamous (*n* = 10), anaplastic (*n* = 2), a high-grade pancreatic intraepithelial neoplasia (*n* = 2), a mixed neuroendocrine non-neuroendocrine neoplasm (*n* = 1) and an unclassifiable neoplasm (*n* = 2). ^‡^ Measured using endoscopic ultrasonography at the first examination and prior to neoadjuvant therapy. Abbreviations: ASA PS, American Society of Anesthesiologist physical status; BMI, body mass index; R, resectable; BR, borderline-resectable; PV, portal vein; A, artery; CA19-9, carbohydrate antigen 19-9; SD, standard deviation; IQR, interquartile range; mod., moderate; PPPD, pylorus-preserving pancreatoduodenectomy; SSPPD, subtotal stomach-preserving pancreatoduodenectomy; Std. PD, standard pancreatoduodenectomy; DP, distal pancreatectomy; TP, total pancreatectomy; mod., moderate; TNM, Tumor–Node–Metastasis.

**Table 2:** Demographic, clinicopathological and treatment characteristics of patients with recurrence.

Variable	ER Group (*n* = 85)	LR Group (*n* = 56)	*p*-Value
Male, *n* (%)	40 (47.1)	33 (58.9)	0.1675
Age, years, mean (SD)	68.0 (9.2)	68.4 (9.1)	0.8119
ASA PS, *n* (%)			0.1981
1–2	75 (88.2)	53 (94.6)	
3	10 (11.8)	3 (5.4)	
BMI, kg/m^2^, mean (SD)	22.4 (3.7)	22.6 (3.4)	0.8200
Resectability, *n* (%)			0.7928
R	60 (70.6)	38 (67.9)	
BR-PV	19 (22.4)	15 (26.8)	
BR-A	6 (7.0)	3 (5.3)	
Preoperative CA19-9 level (U/mL) *			
Median (IQR)	151.1 (45.6–314.6)	75.5 (29.8–244.0)	0.1733
Postoperative CA19-9 level (U/mL) *			
Median (IQR)	27.1 (13.5–108.2)	14.7 (8–32)	0.0113
Surgical procedure, *n* (%)			0.1200
PPPD	9 (10.6)	14 (25.0)	
SSPPD/Std. PD	45 (52.9)	24 (42.9)	
DP	27 (31.8)	17 (30.3)	
TP	4 (4.7)	1 (1.8)	
Vascular resection, *n* (%)	35 (41.2)	19 (33.9)	0.3863
Residual tumor, *n* (%)			0.8852
R0	78 (91.8)	51 (91.1)	
R1/2	7 (8.2)	5 (8.9)	
Histological type, *n* (%)			0.0360
Well-mod. Adenocarcinoma	63 (74.1)	51 (91.1)	
Poor adenocarcinoma	13 (15.3)	2 (3.6)	
Other ^†^	9 (10.6)	3 (5.3)	
Tumor size, cm, mean (SD) ^‡^	2.6 (0.9)	2.3 (0.9)	0.1876
Pathological tumor size, cm, mean (SD)	3.5 (1.8)	3.1 (1.2)	0.1572
T-stage, *n* (%)			0.4509
1–2	67 (78.8)	47 (83.9)	
3	18 (21.2)	9 (16.1)	
Positive lymph nodes, *n* (%)	67 (78.8)	39 (69.6)	0.2169
Perineural invasion, *n* (%)	79 (92.9)	52 (92.9)	0.9848
Lymphovascular invasion, *n* (%)	62 (72.9)	31 (55.4)	0.0311
Venous invasion, *n* (%)	62 (72.9)	37 (66.1)	0.3828
TNM stage, *n* (%)			0.3453
1	17 (20.0)	15 (26.8)	
2	29 (34.1)	22 (39.3)	
3	25 (29.4)	15 (26.8)	
4	14 (16.5)	4 (7.1)	
Neoadjuvant therapy, *n* (%)			0.8380
None	65 (76.4)	45 (80.4)	
Chemotherapy	10 (11.8)	6 (10.7)	
Chemoradiotherapy	10 (11.8)	5 (8.9)	
Adjuvant therapy, *n* (%)			0.5223
None	7 (8.2)	2 (3.6)	
Chemotherapy	77 (90.6)	53 (94.6)	
Chemoradiotherapy	1 (1.2)	1 (1.8)	

* Patients (*n* = 121) had perioperative CA19-9 measurements available. Patients (*n* = 20) with CA19-9 levels < 3 U/mL (related to Lewis-negative patients) were excluded. ^†^ Other consisted of histologic types such as adenosquamous (*n* = 10), a mixed neuroendocrine non-neuroendocrine neoplasm (*n* = 1) and an unclassifiable neoplasm (*n* = 1). ^‡^ Measured using endoscopic ultrasonography at the first examination and prior to neoadjuvant therapy. Abbreviations: ER, early recurrence; LR, late recurrence; ASA PS, American Society of Anesthesiologist physical status; BMI, body mass index; R, resectable; BR, borderline-resectable; PV, portal vein; A, artery; CA19-9, carbohydrate antigen 19-9; SD, standard deviation; IQR, interquartile range; PPPD, pylorus-preserving pancreatoduodenectomy; SSPPD, subtotal stomach-preserving pancreatoduodenectomy; Std. PD, standard pancreatoduodenectomy; DP, distal pancreatectomy; TP, total pancreatectomy; mod., moderate; TNM, Tumor–Node–Metastasis.

**Table 3:** Univariate and multivariate analyses of postoperative risk factors for an early recurrence after a resection.

**Preoperative Risk Factors**	**Univariate**		**Multivariate**	
	**OR (95% CI)**	***p*-Value**	**OR (95% CI)**	***p*-Value**
Age (>70 vs. ≤70 years)	0.95 (0.53–1.69)	0.854	–	–
Sex (male vs. female)	0.88 (0.49–1.56)	0.654	–	–
Resectability (BR vs. R)	1.39 (0.73–2.63)	0.314	–	–
Tumor size (>3.0 vs. ≤3.0 cm) *	3.05 (1.37–6.77)	0.0061	3.11 (1.35–7.14)	0.0076
Tumor location (head/uncinate vs. body/tail)	1.01 (0.54–1.88)	0.983	–	–
Preoperative CA19-9 level (>52 vs. ≤52 U/mL)	3.30 (1.76–6.19)	<0.001	3.25 (1.67–6.30)	<0.001
Neoadjuvant therapy (yes vs. no)	1.19 (0.61–2.33)	0.615	–	–
**Postoperative Risk Factors**	**Univariate**		**Multivariate**	
	**OR (95% CI)**	***p*-Value**	**OR (95% CI)**	***p*-Value**
Age (>70 vs. ≤70 years)	0.95 (0.53–1.69)	0.854	–	–
Sex (male vs. female)	0.88 (0.49–1.56)	0.654	–	–
Tumor size (>3.0 vs. ≤3.0 cm)	2.66 (1.47–4.84)	0.0013	2.00 (1.03–3.90)	0.0420
Tumor differentiation (poor vs. others)	4.38 (1.57–12.24)	0.0049	2.32 (0.75–7.22)	0.1457
Positive lymph nodes (yes vs. no)	2.61 (1.36–5.02)	0.0041	1.62 (0.75–3.47)	0.2184
Distant metastasis (yes vs. no)	3.36 (1.24–9.09)	0.0173	1.66 (0.55–4.98)	0.3651
Perineural invasion (yes vs. no)	3.16 (1.16–8.63)	0.0250	1.43 (0.48–4.25)	0.5213
Lymphovascular invasion (yes vs. no)	2.86 (1.54–5.32)	<0.001	1.74 (0.85–3.56)	0.1282
Venous invasion (yes vs. no)	1.55 (0.83–2.91)	0.1665	–	–
Postoperative CA19-9 level (>37 vs. ≤37 U/mL)	3.18 (1.63–6.23)	<0.001	2.11 (1.02–4.36)	0.0444
Adjuvant therapy (yes vs. no)	2.19 (0.78–6.13)	0.137	–	–

* Measured using endoscopic ultrasonography at the first examination and prior to neoadjuvant therapy. Abbreviations: CA19-9, carbohydrate antigen 19-9; CI, confidence interval; BR, borderline-resectable; OR, odds ratio; R, resectable.

## Data Availability

Data sharing is not applicable to this article. The authors presented all the necessary information in the manuscript.
